# Reversible Stabilization of Nanofiber-Polyplexes through Introducing Cross-Linkages

**DOI:** 10.3390/jfb15010014

**Published:** 2023-12-30

**Authors:** Ryuta Aono, Kenta Nomura, Eiji Yuba, Atsushi Harada

**Affiliations:** 1 Department of Applied Chemistry, Graduate School of Engineering, Osaka Prefecture University, 1-1 Gakuen-cho, Sakai 599-8531, Osaka, Japan; 2 Department of Applied Chemistry, Graduate School of Engineering, Osaka Metropolitan University, 1-1 Gakuen-cho, Sakai 599-8531, Osaka, Japan

**Keywords:** gene delivery, polyplex, reversible stabilization, cross-linking, disulfide bond

## Abstract

Non-viral gene delivery systems are typically designed vector systems with contradictory properties, namely sufficient stability before cellular uptake and instability to ensure the release of nucleic acid cargoes in the transcription process after being taken up into cells. We reported previously that poly-(L-lysine) terminally bearing a multi-arm PEG (maPEG-PLL) formed nanofiber-polyplexes that suppressed excessive DNA condensation via steric repulsion among maPEGs and exhibited effective transcriptional capability in PCR amplification experiments and a cell-free gene expression system. In this study, the reversible stabilization of a nanofiber-polyplex without impairing the effective transcriptional capability was investigated by introducing cross-links between the PLL side chains within the polyplex using a cross-linking reagent with disulfide (SS) bonds that can be disrupted under reducing conditions. In the presence of dextran sulfate and/or dithiothreitol, the stability of the polyplex and the reactivity of the pDNA were evaluated using agarose gel electrophoresis and real-time PCR. We succeeded in reversibly stabilizing nanofiber-polyplexes using dithiobis (succinimidyl propionate) (DSP) as the cross-linking reagent. The effect of the reversible stabilization was confirmed in experiments using cultured cells, and the DSP-crosslinked polyplexes exhibited gene expression superior to that of polyethyleneimine polyplexes, which are typical polyplexes.

## 1. Introduction

A major bottleneck in non-viral gene delivery systems is the stability of the nanocarriers containing nucleic acid in the blood. After intravenous administration, the nanoparticles must evade extracellular barriers such as nucleases and the reticuloendothelial system until delivery to the target tissue. Unwanted disintegration of the nanocarriers and the subsequent release of the nucleic acids outside the target tissue may cause unintended side effects because of the nanocarrier components and loss of the expected pharmacological effect of the nucleic acid [1,2,3,4,5,6]. Numerous studies on the stability of nanocarriers in blood have been conducted, with most examining the modification of nanoparticles with hydrophilic and flexible polymers (e.g., PEG). Introducing PEGylated lipids or copolymers containing PEG chains into nanocarriers is a gold standard to shield their surface properties and inhibit nonspecific interactions with blood components, which extends their blood circulation time [7,8,9,10]. Conversely, PEGylaytion reduces the efficient uptake of nanocarriers by target cells and the subsequent release of nucleic acid into the cytosol and is termed the PEG dilemma [11,12,13]. Therefore, an advanced carrier design that overcomes this trade-off has long been desired.

Tumor tissues generally have an acidic pH and a high concentration of glutathione (GSH) in the cytosol compared to normal cells. In addition, specific enzymes and receptors are overexpressed in tumor tissues. Thus, many studies have been conducted on stimuli-responsive nanocarriers that leverage this unique environment [14,15,16,17,18]. Ideal stimuli-responsive nanocarriers form stable complexes during blood circulation. However, after accumulating in tumor tissues or intracellular transfer, these stimuli-responsive nanocarriers become unstable in response to environmental stimuli, leading to the release of their nucleic acid cargoes. Examples that have taken advantage of the abundant GSH in the cytoplasm of cancer cells include reversible stabilization by introducing diselenide (SeSe), succinimide-sulfide bonds or disulfide (SS) bonds into nanocarriers [19,20,21,22,23,24,25]. Redox regulation in cancer cells involves primarily the reversible redox reaction of thiol groups on cysteine residues, and various cysteine-containing oxidoreductases, such as thioredoxin and glutaredoxin, regulate the reductive state of the cytosol. Among them, glutathione is particularly abundant in cells, and reduced GSH and oxidized (glutathione disulfide, GSSG) forms are the most important redox pair in the intracellular reducing environment. The intracellular GSH concentration is ~100–1000 fold higher than outside cancer cells ([GSH]_intra_ = 1–11 mM, [GSH]_extra_ = 10 µM). Thus, the intracellular environment exhibits a more reductive environment than the extracellular environment [22,23]. Redox-responsive drug delivery platforms that control the stability of nanocarriers by cleaving the SS bonds introduced between polymer chains in response to the high level of GSH in cancer cells have been investigated extensively. The SS bond is mainly introduced by oxidizing thiol groups that are inherently present in the chosen molecule or by using a cross-linking reagent with a disulfide bond. The former method requires a two-step reaction, whereas the latter introduces an SS bond into the molecule in a single step [23]. Dithiobis (succinimidyl propionate) (DSP) and dimethyl 3,3′-dithiobispropionimidate (DTBP), as amine-reactive cross-linking reagents with a disulfide bond, and disuccinimidyl suberate (DSS) and dimethyl suberimidate (DMS), as their counterparts that do not contain disulfide bonds, are used mainly in protein structural analyses. In nanomedicine, successful reversible stabilization of polyplexes, which were prepared using poly(L-lysine) and polyethyleneimine, via introducing DSP or DTBP has been reported [26,27,28,29,30,31].

**Scheme 1:**
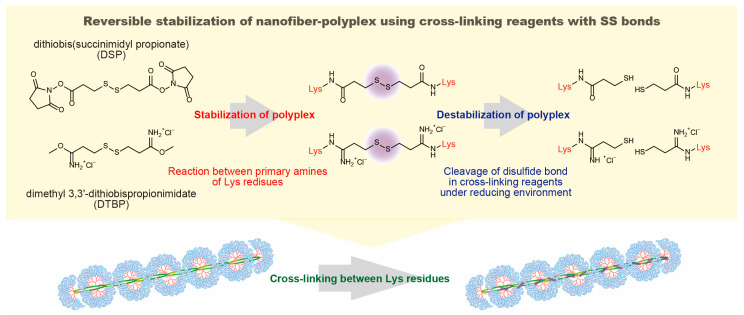
Reversible stabilization of nanofiber-polyplexes using cross-linking reagents with disulfide bonds.

We have previously shown that a head–tail-type polycation composed of poly(L-lysine) terminally bearing multi-arm PEG (maPEG-PLL) suppresses excessive DNA condensation during polyplex formation via steric repulsion of maPEGs and exhibits effective transcriptional capability in a cell-free gene expression system [32,33,34,35]. Unfortunately, the cellular uptake efficiency of maPEG-PLL polyplexes is low because of the elongated nanofiber-like morphology induced by the steric repulsion of intrapolyplex maPEGs [32,33,34]. Therefore, the aim of the present study was to assess the effects of the reversible stabilization, morphology, and gene expression efficiency of the maPEG-PLL polyplex via the introduction of SS bonds using DSP and DTBP as cross-linking reagents (Scheme 1).

## 2. Materials and Methods

### 2.1. Materials

The maPEG-PLL (number of PEG arms = 16, Mn of PEG = 5000 and number-averaged polymerization degree of PLL = 71) was synthesized as described previously [35]. Briefly, the PLL part was polymerized from the focal point of a polyamidoamine (PAMAM) dendron after synthesizing the PAMAM dendron and introducing PEG chains at its peripheral termini. The dithiobis (succinimidyl propionate) (DSP), dimethyl 3,3′-dithiobispropionimidate (DTBP), disuccinimidyl suberate (DSS) and dimethyl suberimidate (DMS) were purchased from Thermo Fisher Scientific Inc. (Tokyo, Japan). The plasmid DNA (pCMV-Luc), which contains the cDNA of firefly luciferase driven by a human cytomegalovirus immediate-early promoter, was amplified in *E. coli*, isolated and purified using a FastGeneTM Plasmid Mini kit (Nippon Genetics, Tokyo, Japan). The agarose and dextran sulfate (sodium salt) were purchased from Nacalai Tesque (Kyoto, Japan). HeLa cell line was obtained from Cell Resource Center for Biomedical Research, Tohoku University (Sendai, Japan). The fetal bovine serum (FBS) was purchased from SAFC Biosciences (Tokyo, Japan). The Dulbecco’s modified Eagle’s medium (DMEM) was purchased from Nissui Pharmaceutical (Tokyo, Japan). The Luc-PGC-50 detergent was purchased from Toyo Ink (Tokyo, Japan). The Coomassie Protein Assay Reagent was purchased from Thermo Fisher Scientific Inc. The IT Fluorescein Labeling Kit was purchased from Mirus (Madison, WI, USA). The linear polyethyleneimine (PEI; Mw 25,000) was purchased from Alfa Aesar (Lancashire, UK).

### 2.2. Preparation of Polyplexes and Introduction of Cross-Linkages

The pDNA and the maPEG-PLL were separately dissolved in phosphate-buffered saline (PBS), and the polyplex solutions were prepared by mixing both solutions at an *N*/*P* ratio = 3, in which the *N*/*P* ratio was defined as the number of primary amines of Lys residues in the maPEG-PLL against the number of phosphates of the pDNA. For the stabilization of the polyplexes, the cross-linking reagents, including DSP, DTBP, DSS and DMS in DMSO, were respectively added to the polyplex solutions at varying molar ratios of cross-linking reagent to Lys residues in the maPEG-PLL ([linker]/[Lys]). The crosslinked polyplexes were stored overnight or longer before the varying evaluation.

### 2.3. Agarose Gel Electrophoresis

The polyplexes with and without cross-linking were added to given amounts of dextran sulfate, in which the number of sulfates was five times that of the phosphates of the pDNA in the solution, and 10 mM dithiothreitol (DTT) dissolved in PBS and incubated at room temperature overnight. The samples (10 μL) were electrophoresed on a 0.6 wt% agarose gel in a 40 mM Tris, 20 mM sodium acetate and 2 mM EDTA buffer (pH 8.0) containing 1 μg/mL ethidium bromide at 100 V for 30 min. The ethidium bromide-stained bands were visualized using a LAS-1000UVmini (Fujifilm, Tokyo, Japan).

### 2.4. Atomic Force Microscopy

For the morphology observation, the DSP- and DTBP-crosslinked polyplexes were prepared at [linker]/[Lys] = 0.2. A drop of the crosslinked or non-crosslinked polyplex solution was placed on freshly cleaved mica, removed via a dry air spray, and allowed to dry. AFM images were collected using an SPA-400 atomic force microscope (Seiko Instruments Inc., Chiba, Japan) in the tapping mode. For each sample, the lengths of the major and minor axes of 100 polyplexes were measured, and the aspect ratios were calculated from the average lengths of the major and minor axes.

### 2.5. Reactivity of Polymerase to pDNA in the Polyplexes

The reactivity of polymerase to the pDNA in the polyplexes were evaluated via real-time PCR measurements using a LightCycler Nano (Roche, Basel, Switzerland), in which the luciferase amplification primers were 5′-GCGCGGAGGAGTTGTGTT-3′ for the forward primer and 5′-TCTGATTTTTCTTGCGTCGAGTT-3′ for the reverse primer [36]. The sample solutions of crosslinked and non-crosslinked polyplexes were prepared and stored with and without sufficient amounts of dextran sulfate, in which the number of sulfates was five times that of the phosphates of the pDNA in the solution, and/or 10 mM DTT. The sample solutions were serially diluted to provide a pDNA copy number of 10^7^–10^4^ copies/5 μL. Five microliters of the diluted sample solution, 10 μL of FastStart Essential DNA Green Master (Roche, Basel, Switzerland), 1 μL of 10 μM forward primer solution, 1 μL of 10 μM reverse primer solution and 3 μL nuclease-free water were mixed, and real-time PCR measurements were performed. The calibration curve between the logarithm of the pDNA copy number (10^4^, 10^4.5^, 10^5^, 10^5.5^, 10^6^, 10^6.5^, 10^7^ copies) and the threshold cycle (*C*t) value was prepared. From the *C*t value of the sample with a pDNA copy number of 10^7^, the corresponding copy number of pDNA was calculated using the calibration curve and the PCR efficiency was then determined as the percentage of the calculated copy number of pDNA against that of 10^7^.

### 2.6. Cellular Uptake and Transfection of the Polyplexes

The cellular uptake was evaluated using FITC-labeled pDNA, which was labeled using the IT Fluorescein Labeling Kit according to the protocol provided by the manufacturer. Non-labeled pDNA was used for the transfection experiment. HeLa cells, which were derived from human cervical cancer, were seeded in 0.5 mL DMEM supplemented with 10% FCS in 24-well culture plates at 5 × 10^4^ cells per well the day before the uptake experiments. The cells were washed with PBS containing 0.36 mM CaCl_2_ and 0.42 mM MgCl_2_ [PBS(+)] and then covered with DMEM (1 mL). The non-crosslinked and DSP-crosslinked polyplex solutions containing pDNA (1 μg) were gently added to the cells and incubated at 37 °C for 24 h. The cells were washed with PBS(+) and detached using trypsin to evaluate the cellular uptake. The cellular fluorescence was then evaluated via flow cytometry (EPICS XL, Beckman Coulter, Inc., Tokyo, Japan). The transfection efficiency of the DSP- or DSS-crosslinked and non-crosslinked polyplexes were evaluated by means of luciferase assay. Additionally, the transfection efficiency was also evaluated for the PEI polyplexes, which were prepared at an *N*/*P* ratio = 10, for the comparison with the representative polyplex. After 24 h of incubation of the HeLa cells with polyplex solutions, the cells were lysed by adding 50 μL of the Luc-PGC-50 detergent. A 20 μL aliquot from each dish was used for one luciferase assay using a kit (Toyo Ink, Tokyo, Japan) and a Lumat LB9507 luminometer (Berthold, Bad Wildbad, Germany). The protein content of the lysate was measured using the Coomassie Protein Assay Reagent and bovine serum albumin as the standard.

### 2.7. Statistical Analysis

Statistically significant differences between the experimental groups were determined using Prism software (v9, GraphPad, Boston, MA, USA). One-way analysis of variance (ANOVA) followed by Tukey’s HSD post hoc test were used. The *p* values are shown in the caption of each experimental result.

## 3. Results and Discussion

**Figure 1:**
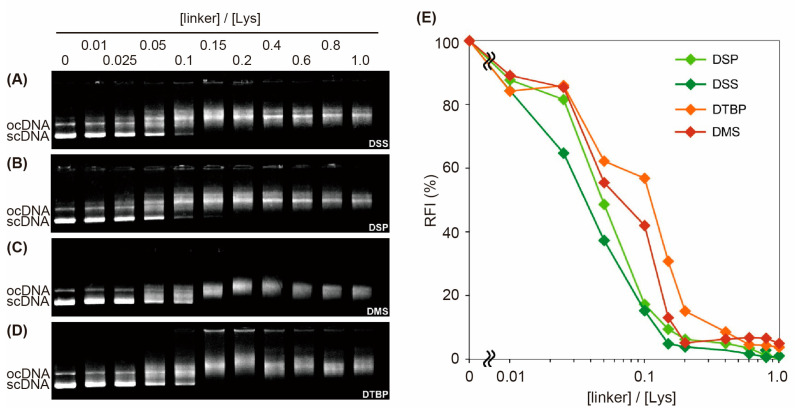
Effect of the amount of cross-linking reagent on stabilizing the polyplexes. Gel electrophoretic images of nanofiber-polyplexes mixed with each cross-linking reagent ((**A**) DSS; (**B**) DSP; (**C**) DMS; (**D**) DTBP) after adding dextran sulfate. Changes in the relative fluorescence intensity (RFI) as the [linker]/[Lys] ratio increases for the cross-linkers DSS, DSP, DMS and DTBP (**E**). The RFI was calculated with the fluorescence intensity at the same migration distance and band area as naked scDNA taken as 100%.

Nanofiber-polyplexes with an elongated morphology generated via the PEG crowding effect were prepared by mixing pDNA and maPEG-PLL, as previously reported [32,34]. The cross-linking reagents DSS, DSP, DMS and DTBP were added to the nanofiber-polyplex solutions at varying [linker]/[Lys] ratios, in which the *N*-hydroxysuccinimide ester reacts with the primary amine of the Lys residue in the case of DSS and DSP, and the imidoester with the primary amine of the Lys residue in the case of DMS and DTBP. The stabilization of PLL polyplexes using the DTBP and PEI polyplexes using DSP and DTBP was confirmed by the change in ethidium bromide (EtBr) fluorescence from the interactions with the PLL/DNA and PEI/DNA polyplexes in the presence of a competing polyanion [poly(L-aspartic acid) or poly(methacrylic acid)] [27,28]. This assay takes advantage of the difficulty of intercalating EtBr into pDNA in the polyplex. However, in the case of nanofiber-polyplexes formed by maPEG-PLL with pDNA, the stabilization generated by introducing cross-linkages cannot be evaluated using the same method because EtBr can intercalate with pDNA even within the nanofiber-polyplex to the same extent as naked pDNA [37]. Here, the stabilization of introducing cross-linkages between the Lys residues in the polyplexes was evaluated using agarose gel electrophoresis after adding dextran sulfate, in which enough dextran sulfate was added to the polyplexes to release pDNA through the polyanion exchange reaction between pDNA and dextran sulfate. The gel electrophoretic images of the nanofiber-polyplexes mixed with each cross-linking reagent after adding dextran sulfate are shown in Figure 1A–D. In all the gel electrophoresis images without a cross-linking reagent ([linker]/[Lys] ratio = 0), the pDNA was completely released from the polyplex by the addition of dextran sulfate, and open circular and supercoiled DNA (scDNA) bands were observed. For each lane in Figure 1A–D, the relative fluorescence intensity (RFI) was calculated with the fluorescence intensity at the same migration distance and band area as naked scDNA taken as 100%. Figure 1E shows the change in the RFI as the [linker]/[Lys] ratio increases when using DSS, DSP, DMS and DTBP as cross-linking reagents. The RFI gradually decreased with increasing [linker]/[Lys] ratios for all the cross-linking reagents examined, demonstrating the inhibition of pDNA release. This inhibition arises from introducing cross-linkages between the PLL chains, which effectively entangle the pDNA and PLL chains in maPEG-PLL, suppressing the polyanion exchange reaction between the pDNA and dextran sulfate. Free scDNA was no longer observed at [linker]/[Lys] ratios of more than 0.2. Interestingly, when the replacement of pDNA and polyanion is suppressed, the pDNA does not migrate because of the charge neutralization caused by the polycation. However, at [linker]/[Lys] ratios of more than 0.2, a migrating band with a shorter migration distance than naked pDNA was observed. The maPEG-PLL that did not effectively entangle with the pDNA was released from the polyplex by adding dextran sulfate, and the maPEG-PLL, in which the PLL chains and DNA were effectively entangled through the reaction with the cross-linking reagents, was maintained in a polyplex with pDNA. Thus, the crosslinked polyplex was negatively charged and migrated in the gel electrophoresis. Crosslinked polyplexes prepared at [linker]/[Lys] ratio = 0.2 were used in the subsequent experiments.

**Figure 2:**
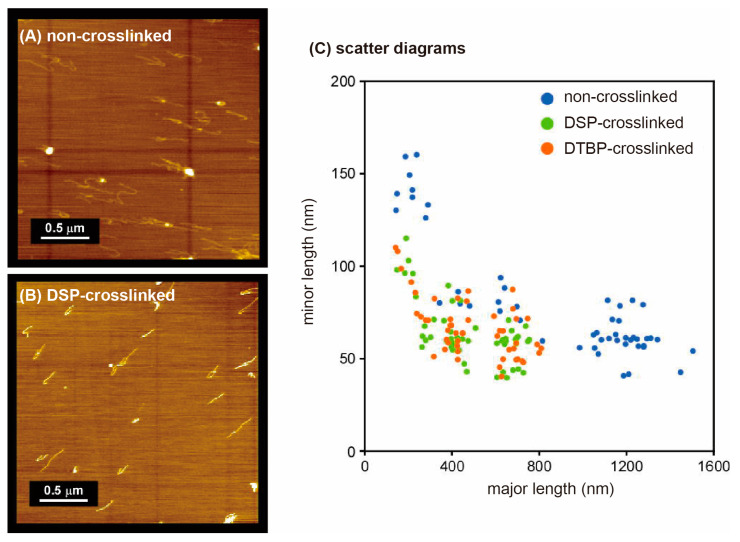
Effect of introducing cross-linkages into the polyplex morphology. AFM images of non-crosslinked (**A**) and DSP-crosslinked (**B**) polyplexes on mica. Scatter diagrams of the long and short axes of polyplexes with/without cross-linking using DSP and DTBP (**C**).

The effect of introducing cross-linkages into the polyplex morphology was evaluated via AFM observation. AFM images for polyplexes without and with cross-linking by DSP are shown in Figure 2A,B. An elongated polyplex morphology, i.e., nanofiber-polyplex, was observed with or without cross-linking. For the PEI polyplexes, introducing cross-linkages using DSP did not influence the polyplex morphology, as observed via AFM [30]. However, induced differences in the degree of morphology elongation for the nanofiber-polyplexes were observed. The long and short axes of each polyplex were measured as indicators of the morphological characteristics, and scatter diagrams of the long and short axes of polyplexes with/without cross-linking were prepared using DSP and DTBP as cross-linking reagents (Figure 2C). Table 1 summarizes the average long axis, short axis, and aspect ratio of the nanofiber-polyplexes. As reported previously [32], in the case of non-crosslinked nanofiber-polyplexes, DNA condensation was suppressed because of the PEG crowding effect, and an elongated morphology with a long axis length of ~1000–1300 nm was observed in the AFM observations. This range of the long axis length is comparable to the length of the twisted pDNA structure used here (approximately 1200 nm; 7 kbp × 0.34 nm/bp/2 = 1190 nm). There was no difference in the distribution of the crosslinked polyplexes when using either the DSP or DTBP cross-linking reagent, and no morphology with a long axis length of more than 1000 nm was observed (Figure 2C). In the crosslinked polyplexes, there were mainly two distributions of major axis lengths: 600–700 nm and 300–400 nm. These distributions correspond to the once-folded (1200/2 nm) and twice-folded (1200/3 nm) structures of fully elongated pDNA. In our previous study, we confirmed that when the PEG crowding effect is reduced, the polyplex with a fully extended pDNA can no longer form, and polyplexes with long axis lengths comparable to once- or twice-folded structures are formed [33]. The result obtained here likely arises from immobilizing the PLL chain of the maPEG-PLL because of the introduction of cross-linkages, which reduced the PEG crowding effect. As a result, the aspect ratio of the crosslinked polyplexes was slightly reduced compared to non-crosslinked polyplexes; nonetheless, the crosslinked polyplexes had relatively elongated morphologies with an aspect ratio of ca. 7 (Table 1).

**Figure 3:**
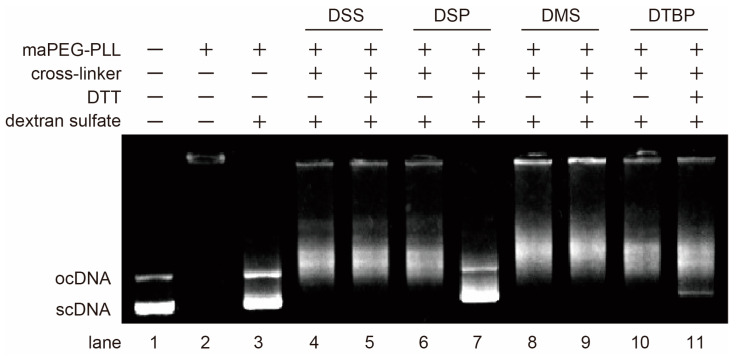
Agarose gel electrophoretic image of polyplexes with or without a cross-linker, DTT and dextran sulfate. lane 1, nacked pDNA; lane 2, nanofiber-polylexes; lane 3, nanofiber-polyplexes with dextran sulfate; lane 4, DSS-crosslinked polyplexes with dextran sulfate; lane 5, DSS-crosslinked polyplexes with dextran sulfate and DTT; lane 6, DSP-crosslinked polyplexes with dextran sulfate; lane 7, DSP-crosslinked polyplexes with dextran sulfate and DTT; lane 8, DMS-crosslinked polyplexes with dextran sulfate; lane 9, DMS-crosslinked polyplexes with dextran sulfate and DTT; lane 10, DTBP-crosslinked polyplexes with dextran sulfate; lane 11, DTBP-crosslinked polyplexes with dextran sulfate and DTT.

The destabilization of the crosslinked polyplexes through cleaving the SS bonds in response to reducing environments was assessed via agarose gel electrophoresis (Figure 3). Comparing lanes 2 and 3 in Figure 3, the non-crosslinked polyplex released pDNA in the presence of dextran sulfate. No scDNA band was observed when crosslinked polyplexes were prepared with the cross-linking reagents (DSS, DSP, DMS and DTBP) (Figure 1), confirming the stabilization by means of the cross-linkages (lanes 4, 6, 8 and 10). In the polyplexes stabilized with cross-linking reagents with no SS bond, there was no change in the migration band caused by adding the reducing agent DTT (lanes 4 and 5 for DSS, lanes 8 and 9 for DMS). That is, the cross-linking of the polyplex using DSS or DMS with no SS bond in the molecule led to irreversible stabilization. In contrast, for polyplexes stabilized via cross-linking reagents with SS bonds (DSP, DTBP), pDNA was released upon adding DTT (lanes 7 and 11). This release of pDNA occurred because the entanglement between the crosslinked PLL chains and DNA is resolved via cleaving the SS bonds. Additionally, more pDNA was released upon DTT addition in the DSP-crosslinked polyplex than in the DTBP-crosslinked polyplex. Differences in the reaction between the cross-linking reagents and Lys residues may be responsible for this observation. As shown in Scheme 1, the reaction of the imidoester of DTBP with a Lys primary amine leads to the loss of the positive charge of the primary amine, although a new positively charged amidine bond is formed. In contrast, Lys residues lose their positive charge when reacting with the *N*-hydroxysuccinimide ester of DSP. As a result, when DSP is used as a cross-linking reagent, the electrostatic interaction of the PLL chains with the pDNA weakens, which facilitates the release of pDNA. Similar results have been reported for DSP-crosslinked PEI polyplexes [28], in which the polyplex stability analyzed via agarose gel electrophoresis with poly(methacrylic acid) and with or without dithioerythritol showed the complete release of pDNA from DSP-crosslinked PEI polyplexes in the presence of poly(methacrylic acid) and dithioerythritol.

**Figure 4:**
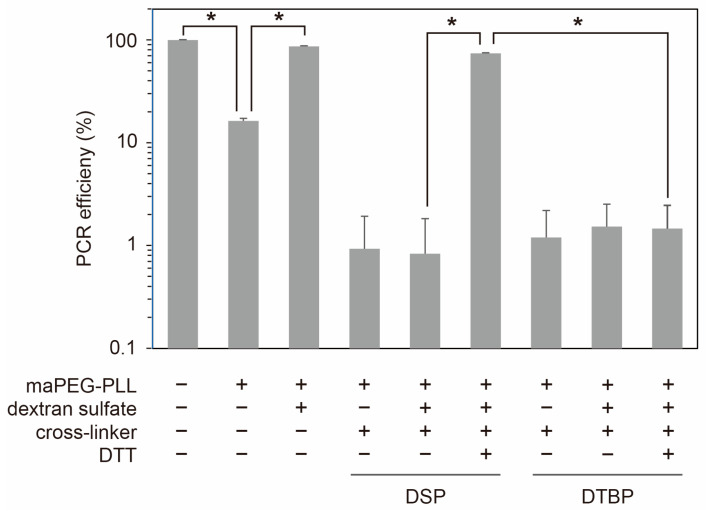
The reactivity of the pDNA in the polyplexes with polymerase was evaluated via real-time PCR under various conditions. The PCR efficiency was determined from the calibration curve of naked pDNA, and the data are presented as the average of three experiments ± SD. The symbols * indicate *p* values less than 0.0001.

Stabilization of the polyplexes by introducing cross-linkages may affect the reactivity of pDNA, including the transcription process. We have previously reported the efficiency of the PCR amplification of pDNA in a polyplex-formed state as an index reflecting the efficiency of the transcription process [33]. Polyplex formation with maPEG-PLL was found to suppress the excessive condensation of pDNA, resulting in extremely higher PCR efficiency than polyplex formation with a PLL homopolymer [33]. We compared the PCR efficiency under various conditions to examine the effect of introducing cross-linkages into polyplexes on pDNA reactivity (Figure 4). As previously reported, the non-crosslinked polyplex showed relatively high PCR efficiency (ca. 15%) as a polyplex. Adding dextran sulfate increased the PCR efficiency to 87% because of the release of pDNA from the polyplex. The PCR efficiency of both the DSP-crosslinked and DTBP-crosslinked polyplexes decreased to just under 1% because of the introduction of cross-linkages. The addition of dextran sulfate did not lead to an increase in the PCR efficiency because no pDNA was released, as shown in Figure 1. The differences between the DSP- and DTBP-crosslinked polyplexes were confirmed when both dextran sulfate and DTT were added. The PCR efficiency of the DSP-crosslinked polyplex recovered to 75% upon adding dextran sulfate and DTT. In contrast, the PCR efficiency of the DTBP-crosslinked polyplex was unchanged in the presence of these two compounds. This observation is consistent with the results evaluating the destabilization of the crosslinked polyplexes in response to reducing environments (Figure 3), indicating that the maPEG-PLL must be completely separated from the pDNA to enable the reaction between the pDNA and polymerase. In summary, the results in Figure 1, Figure 3 and Figure 4 show that using DSP with an SS bond as the cross-linking reagent can provide a reversibly stable nanofiber-polyplex.

**Figure 5:**
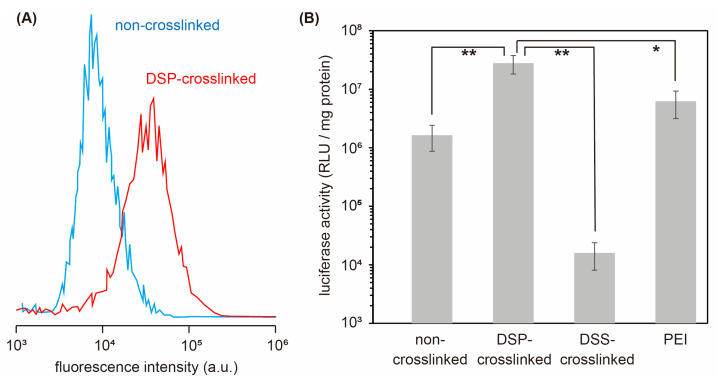
The in vitro performance of the polyplexes with and without cross-linkages. Cellular uptake of DSP-crosslinked and non-crosslinked polyplexes evaluated via flow cytometry (**A**). Luciferase activity of HeLa cells treated with non-crosslinked, DSP-crosslinked, DSS-crosslinked polyplexes and PEI polyplexes (**B**). The data in (**B**) are presented as the average of three experiments ± SD. The symbols * and ** indicate *p* values less than 0.05 and 0.01, respectively.

Finally, the effect of the reversible stabilization of nanofiber-polyplexes on the cellular uptake and transfection was examined against cultured cells (HeLa cells). Using FITC-labeled pDNA, the cellular uptake of the DSP-crosslinked polyplexes and non-crosslinked polyplexes was examined by means of flow cytometry (Figure 5A). The average fluorescence intensity of the cells treated with DSP-crosslinked polyplexes was 32,000, compared to 8400 for the cells treated with non-crosslinked polyplexes. Also, the percentages of positive cells compared with non-treated cells were 91% and 100% for the cells treated with non-crosslinked polyplexes and DSP-crosslinked polyplexes, respectively. This increase in the cellular fluorescence intensity arises from the stabilization of the polyplexes by cross-linking and the slight decrease in the aspect ratio of the polyplexes summarized in Table 1. The cellular uptake of polyplexes elongated by the PEG crowding effect was dependent on the aspect ratio of the polyplexes, with the cellular uptake of longer elongated polyplexes, i.e., nanofiber-polyplexes, hampered. The transfection efficiency of the DSP- and DSS-crosslinked polyplexes and non-crosslinked polyplexes was evaluated using the luciferase assay (Figure 5B). The reversibly stabilized DSP-crosslinked polyplexes exhibited remarkably high transfection efficiency compared to the irreversibly stabilized DSS-crosslinked and non-crosslinked polyplexes. The release of pDNA in the DSS-crosslinked polyplexes was not observed even in the presence of dextran sulfate and DTT (Figure 3). Therefore, the transfection efficiency was reduced by introducing DSS cross-linkages without SS bonds compared to the non-crosslinked polyplexes. In contrast, the DSP-crosslinked polyplexes exhibited a transfection efficiency two orders of magnitude higher than the DSS-crosslinked polyplexes and more than one order of magnitude higher than the non-crosslinked polyplexes. Non-crosslinked polyplexes have been reported to escape from endosomes because of the buffering effect of the tertiary amines in the interior of the PAMAM dendron, with no influence of chloroquine on their transfection level [33]. The DSP-crosslinked polyplexes showed an effective buffering effect for endosomal escape. The DSP-crosslinked polyplexes were likely destabilized through SS bond cleavage in an intracellular reducing environment and transformed into polyplexes that exhibit high transcription efficiency. The transfection efficiency level of the DSP-crosslinked polyplexes also exceeded that of the PEI polyplexes, which are the representative polyplex.

## 4. Conclusions

In this report, the reversible stabilization of nanofiber-polyplexes formed from pDNA and maPEG-PLL without compromising their high transcription efficiency properties was investigated by comparing cross-linking reagents with and without an SS bond in the molecule. The stability of the polyplexes in the presence of dextran sulfate, a polyanion, was improved in the case using both a cross-linking reagent with and without an SS bond, and the stabilizing effect was eliminated only by using a cross-linking reagent with an SS bond in the molecule that cleaved under reducing conditions, e.g., in the presence of DTT. It was demonstrated that the use of a cross-linking reagent with an SS bond could provide the reversible stabilization of the nanofiber-polyplexes. Because of this reversible stabilization, the nanofiber-polyplexes crosslinked by DSP bearing an SS bond in the molecule exhibited higher gene expression than conventional PEI polyplexes. Introduction of cross-linkages into polyplexes using the reagents with SS bonds within the molecule can stabilize the polyplexes without compromising the properties of the polyplex before cross-linking and allow destabilization through the cleavage of SS bonds in a cytoplasmic reducing environment, and this reversible stabilization is applicable to polyplexes using other polycations for improving the gene expression.

## Figures and Tables

**Table 1:** Major and minor axis lengths of polyplexes with/without introducing cross-linkages.

Type of Polyplexes	Length (nm ± SD) ^1^		Aspect Ratio (L_maj_/L_min_)
	Major (L_maj_)	Minor (L_min_)	
Non-crosslinked	799 ± 395	80 ± 32	10.01
DSP-crosslinked	457 ± 171	64 ± 17	7.12
DTBP-crosslinked	460 ± 177	67 ± 16	6.91

^1^ The axis length of 100 polyplexes for each type of polyplex was measured using AFM images.

## Data Availability

Data are contained within the article.
